# Standardization of the tumor-stroma ratio scoring method for breast cancer research

**DOI:** 10.1007/s10549-022-06587-3

**Published:** 2022-04-16

**Authors:** Sophie C. Hagenaars, Kiki M. H. Vangangelt, Gabi W. Van Pelt, Zsófia Karancsi, Rob A. E. M. Tollenaar, Andrew R. Green, Emad A. Rakha, Janina Kulka, Wilma E. Mesker

**Affiliations:** 1 Department of Surgery, Leiden University Medical Centre, Albinusdreef 2, 2333 ZA, Leiden, The Netherlands; 2 2Nd Department of Pathology, Semmelweis University, Budapest, Hungary; 3 Academic Unit for Translational Medical Sciences, School of Medicine, Nottingham Breast Cancer Research Center, The University of Nottingham, Nottingham, UK

**Keywords:** Breast cancer, Tumor microenvironment, Biomarker, Tumor-stroma ratio, Protocol, Artificial intelligence

## Abstract

**Purpose:**

The tumor-stroma ratio (TSR) has repeatedly proven to be correlated with patient outcomes in breast cancer using large retrospective cohorts. However, studies validating the TSR often show variability in methodology, thereby hampering comparisons and uniform outcomes.

**Method:**

This paper provides a detailed description of a simple and uniform TSR scoring method using Hematoxylin and Eosin (H&E)-stained core biopsies and resection tissue, specifically focused on breast cancer. Possible histological challenges that can be encountered during scoring including suggestions to overcome them are reported. Moreover, the procedure for TSR estimation in lymph nodes, scoring on digital images and the automatic assessment of the TSR using artificial intelligence are described.

**Conclusion:**

Digitized scoring of tumor biopsies and resection material offers interesting future perspectives to determine patient prognosis and response to therapy. The fact that the TSR method is relatively easy, quick, and cheap, offers great potential for its implementation in routine diagnostics, but this requires high quality validation studies.

## Introduction

Over the last decade, the tumor microenvironment (TME) has shown to be an important factor in the prognostication of epithelial tumors [1–4]. The tumor-stroma ratio (TSR), which is the proportion of tumor-related stroma within a malignancy scored per tenfold percentage, may be the microscopically visible, simplified translation of the complex biological process of tumor cell-tumor microenvironment interactions. The TSR is typically assessed on conventional Hematoxylin and Eosin (H&E)-stained slides of resection material or biopsies from the primary tumor [5]. Studies have repeatedly shown that the TSR is correlated to clinicopathological parameters and that tumors with a high amount of stroma are associated with a worse prognosis compared to stroma-low tumors [6]. This resulted in the TSR being identified as a potentially new parameter for routine histological evaluation for patient prognosis. The inferior outcome of stroma-high tumors was not only shown in breast cancer [4, 7–13], but also in several other types of cancer, including colon [14–19], cervical [20–22], esophageal [23, 24], and non-small cell lung cancer [25–27]. These studies were performed by various research groups and all confirmed the same associations; however, there are variations in the performance of the method, thereby limiting uniform outcomes.

Various factors that are related to the tumor stroma, such as the dominant stroma type of a tumor (e.g., collagenized cell-poor stroma, fibroblast/myofibroblast-rich stroma, elastotic stroma or tumor-infiltrating lymphocytes (TILs)-rich stroma) [28], stromal organization within the tumor [29], and the density and stiffness of the extracellular matrix [30], have shown to be prognostic factors, related with the response to chemotherapy and predictive for tumor aggressiveness, respectively. However, the exact biological explanation underlying the TSR has not yet been unraveled. Although there is an overall strong association between breast cancer histological type and grade, and the amount and type of stroma, and considering that the ‘no special type’ (NST) breast carcinomas are the most common type (accounting for approximately 75% of cases), the results of the TSR can be applied to breast cancer as a whole, but with some caveats related to some special type tumors, such as invasive lobular carcinomas and mucinous carcinomas.

The technique of visually determining the TSR, which is simple, inexpensive, and fast (generally takes less than 2 min per tissue slide), can easily be implemented in routine diagnostics. Moreover, this method has proven to be highly reproducible for all tumor types, according to an overview of interobserver (kappa) scores ranging from 0.68 to 0.97 of studies which have been executed between 2009 and 2017 [11]. For breast cancer specifically, the Cohen’s kappa coefficient for TSR assessment of resection material ranged from 0.68 to 0.87, showing a reasonably good to very good interobserver agreement (Table 1). The importance of the TSR has gained attention with the introduction of whole slide imaging (WSI) technology in routine practice with the applications of image analysis and artificial intelligence (AI) tools to prognostically classify tumors based on their morphological variables [31, 32], including the TSR [33].

**Table 1:** All breast cancer studies including TSR assessments of tumor resection material with interobserver agreement values and relevance

Study	Number of patients (N)	Interobserver score (κ value)	Relevance of the TSR
De Kruijf EM et al*.* [4]	574	0.85	Prognostic (primary operable BC)
Moorman AM et al*.* [7]	124	0.74	Prognostic (TNBC)
Dekker TJ et al*.* [8]	403	0.804	Prognostic (node-negative BC)
Downey CL et al*.* [48]	63 (subset)	0.70	Prognostic (ER-positive BC)
Roeke T et al*.* [9]	737	0.68	Prognostic (primary operable BC)
Vangangelt KMH et al*.* [10]	344	0.85	Prognostic (primary operable BC combined with immune status)
Vangangelt KMH et al*.* [11]	191	0.85	Prognostic (BC with positive axillary nodes)
Vangangelt KMH et al*.* [49]	619	0.77	Amount of BC stroma increases with age
Vangangelt KMH et al. [12]	1794	0.87	Prognostic (primary operable BC)
Xu Q et al*.* [13]	240	0.77	Prognostic (invasive BC)
Zakhartseva LM et al*.* [50]	232	0.84	Prognostic (primary operable BC)

*BC* breast cancer, *TNBC* triple-negative breast cancer, *ER* estrogen receptor

Based on our experiences with the UNITED study, an international validation study of the TSR for colon cancer [34], and the corresponding E-learning method which was provided to participating pathologists for training purposes, we noticed that clear instructions are essential to meet the study goals for TSR scoring [35]. Consequently, both a uniform TSR scoring method for research purposes and implementation in daily routine pathological diagnostics demand a clear guidance to assure high concordance between observers. An overview of the recommendations for TSR assessment in colon cancer has already been published by our group [11]. However, additional histological difficulties can occur in breast cancer in comparison to colon cancer. Therefore, in this paper, we provide the best practice recommendations for TSR assessment in breast cancer including detailed protocols for future uniform scoring in research and in routine practice, based on scientific data from previous validation studies. Hereby, the TSR can possibly contribute to better prognostication and patient selection for treatment.

## Method

The TSR is based on the determination of the amount of tumor stroma in the primary breast tumor or lymph node metastasis. Scoring can either be performed on resection material or on biopsy tissue. Slides can be evaluated using either conventional TSR assessment, which involves conventional microscopy, or they can be assessed by scoring the TSR digitally on scanned tissue slides. Here, we describe the steps in the process of assessing the TSR.

### Slide selection and origin of material

For resection material of breast tumors, all available H&E-stained histological slides of the primary tumor or metastatic lymph node can be used for scoring. Since breast cancers are often heterogeneous and stromal areas can be present throughout the tumor in a variable proportion, the tissue section with the highest amount of stroma should be selected. Although this may be more time-consuming in case of assessment by eyeballing, it can be performed in a more standardized and objective manner with AI-based tools.

The slide with the highest amount of stroma has shown to be decisive for the final assessment of the percentage of stroma [4]. In case of core biopsy specimens, it is also advised to examine all samples, because of the heterogeneity in stromal percentage that can occur between several biopsies of the same tumor. Tissue microarrays are not suitable for TSR scoring, since these cores are too small for evaluation of both the tumor and its stroma to assess the ratio given the fact that these cores are typically sampled from tumor-rich areas, mainly for the assessment of tumor-related markers. Therefore, the chances are high that the small cores are not representative for the entire tumor in this respect.

### Microscopic TSR scoring procedure

**Fig. 1:**
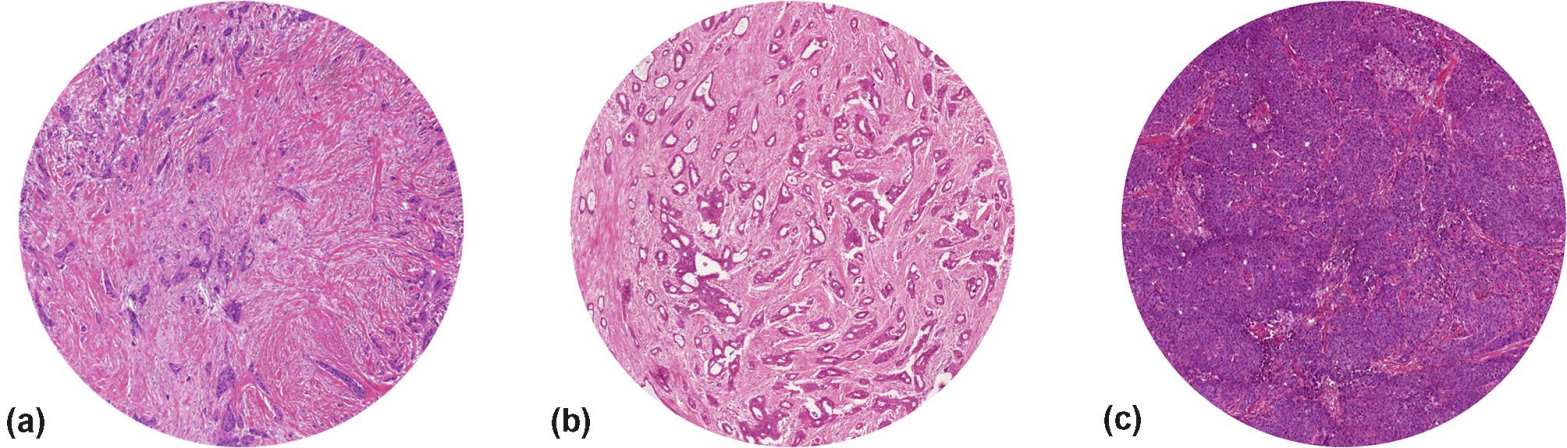
Examples of digitized tissue slides of a stroma-high (80% stroma) primary breast carcinoma (**a**), an intermediate (50% stroma), but still stroma-low tumor (**b**), and a stroma-low (10% stroma) primary breast carcinoma (**c**) which all meet the criterium for correct scoring of tumor cells being present on all four sides of the circular field of vision (field view with × 10 objective)

**Fig. 2:**
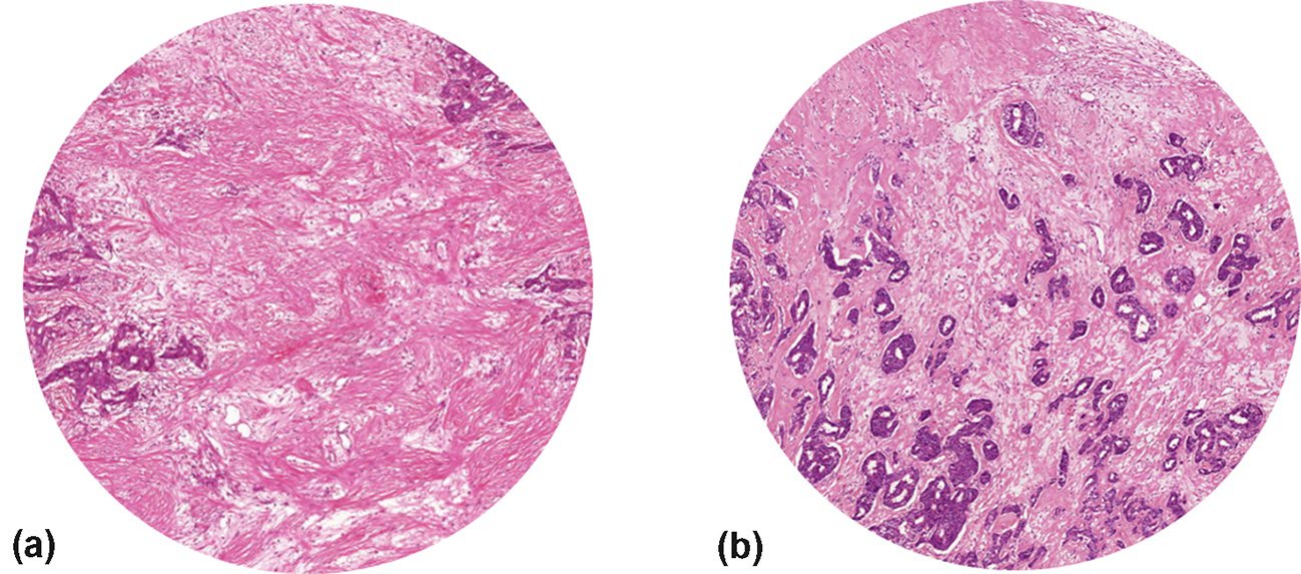
Digitized images of primary tumor tissue sections with tumor cells only present at two (**a**) or three (**b**) borders of the field of vision, therefore, invalid for correct assessment of the TSR on resection specimens (field view with × 10 objective)

For microscopic analysis, routine H&E-stained 5 μm tissue sections cut from formalin-fixed paraffin-embedded (FFPE) blocks of the untreated primary tumor (biopsy and resection) or lymph node metastasis are used for the visual estimation of the TSR. First, a 5 × objective is used to select the most stroma abundant area within the tumor by visually eyeballing the whole tissue slide. Hereafter, the 10 × objective is used to assess the percentage of stroma. The use of a higher magnification results in a non-representative area of the tumor to be evaluated [36]. Furthermore, the microscopic field of vision should contain tumor cells on all four sides of the image (Fig. 1), in order to ensure that only tumor stroma is analyzed, instead of supportive stroma. If the image field does not meet this requirement, for example when only two or three sides of the field of vision contain tumor cells (Fig. 2) or if there is no tumor present at all, another field of vision should be selected.

Determination of the amount of stroma is estimated per 10% increment. A cut-off value of 50% is discriminative for prognosis between stroma-low and stroma-high tumors [5, 8]. Consequently, a stroma-low tumor is defined as a tumor with ≤ 50% stroma (thus TSR high) and a tumor with > 50% stroma is considered stroma-high (thus TSR low). In the learning phase of the TSR scoring method, the TSR of 30% of the slides is ideally scored by two observers. In case of discordance between their scores, a third observer should be consulted.

### Different sizes of oculars

Microscopes used for the conventional scoring method may contain different ocular lenses, with diameters ranging from 1.8 mm to 2.2 mm [11]. Slight differences in ocular lens size should not result in large variances in scoring outcome, but it may lead to different components of the stromal compartment being less well-recognized if lenses deviate considerably from the commonly used range.

### Digital TSR scoring procedure

Digital scoring of the TSR can be performed using an appropriate slide viewer application that is compatible with the image format and the type of scanner. This can be performed by eyeballing using an image analysis tool, in which case it is preferred to use a fixed area on the slide. After scanning the original H&E-stained tissue sections, the amount of stroma can be determined using the predefined annotation with an area size between 2.54 mm^2^ and 3.80 mm^2^, corresponding to the size of the 10 × objective image field of conventional microscopes, of which the diameters ranging from 1.8 mm to 2.2 mm can be considered sufficient.

If there is a possibility of setting a fixed circular area size or diameter in the slide viewer application, a field of 3.46 mm^2^ is preferred, since this annotation size is best comparable with the field of vision of the most frequently used ocular with conventional microscopy. Similar to the microscopic TSR scoring, tumor cells have to be present at all borders of the annotation [36].

### Assessment of TSR on core needle biopsies

**Fig. 3:**
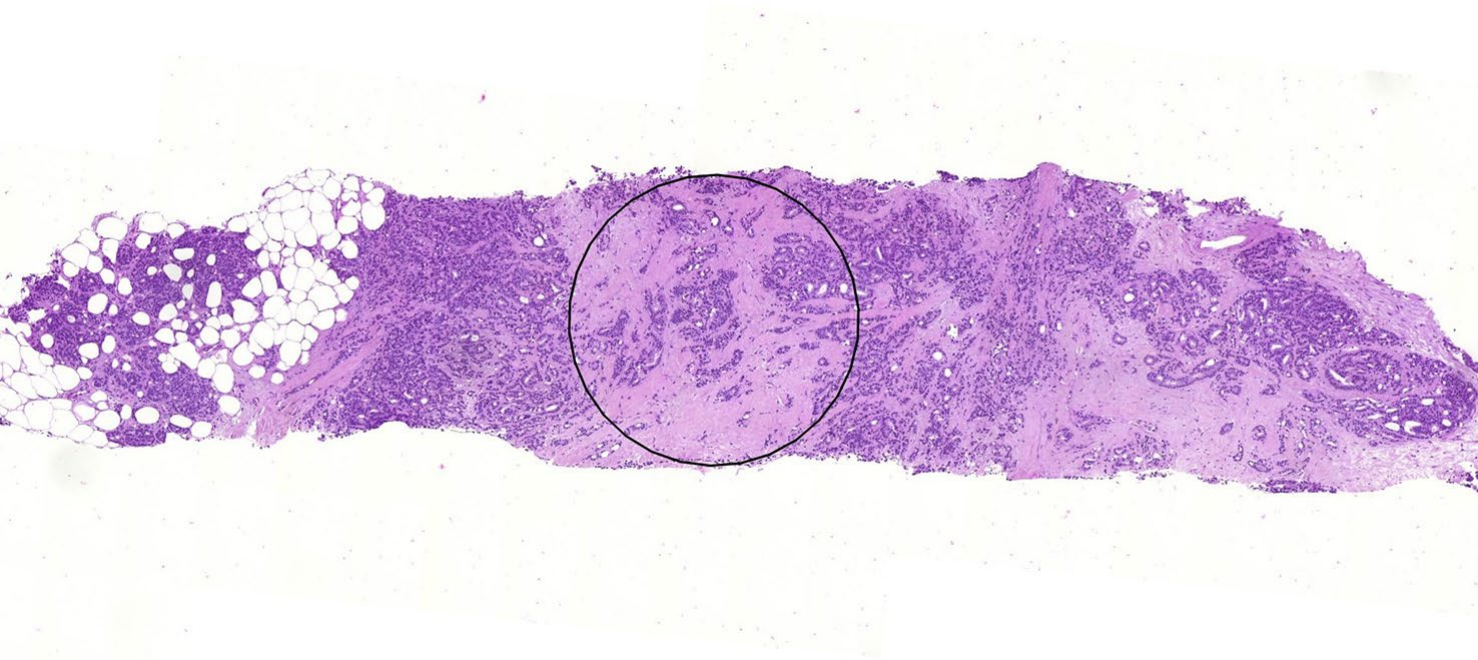
Digitized tissue slide showing an example of an adjusted circle (0.51 mm^2^) to score the TSR on biopsy material (× 10 field view with digital microscope)

Compared to resection material, the diameter of the circular microscopical image field is often larger than that of the core biopsy. As a result, the main rule that tumor cells have to be present at all four borders of the field of vision, is not always completely applicable. Consequently, the TSR can be estimated provided that the borders of the core biopsy contain tumor cells, in addition to two sides of the vision-site. In case of digital TSR assessment, one may reduce the area of the circle to the maximum diameter of the biopsy to display the TSR (Fig. 3), but, preferentially, to apply the standard circle size used for resection material and to visually exclude the parts of the circle that do not include biopsy tissue. Looking at the entire core biopsy is advised while assessing the TSR, but the final score determined based on the circular area is necessary to provide a reference to appropriately compare the TSR scores of different biopsies.

### Assessment of lymph nodes

TSR scoring of H&E-stained lymph node metastases can be carried out in a similar fashion to the procedure for primary tumors. However, if micrometastases are encountered, which are defined as metastases of > 0.2 mm, but ≤ 2 mm, it is allowed to use a smaller image field for the assessment of the TSR, on the condition that this field of vision includes tumor cells at all four borders [11]. In case several lymph nodes are assessed for one patient, the one with the highest stromal percentage is decisive.

### Automatic assessment using AI tools

The majority of previous publications that included the TSR either assessed the tissue slides using a microscope or mimicked the microscopical view during the scoring of digital images. However, in case of automated assessment of the TSR using AI tools or AI-based algorithms on WSI, it is possible to assess the TSR in bigger, possibly even more representative areas, therefore, not only restricting to the small area used by microscopes. Moreover, AI tools are increasingly used in routine practice in addition to research settings, for instance for patient stratification and selection [31, 32]. Therefore, AI tools for the automated assessment of the TSR for breast cancer are currently being developed.

## Histological difficulties in TSR scoring

The TME consists of stromal cells, mainly fibroblasts and myofibroblasts, the extracellular matrix including stromal fibers and ground substance, in addition to other components, such as blood vessels, nerves, inflammatory and immune cells [1]. For large blood vessels and infiltration with inflammatory cells, specific rules apply for TSR assessment, which could influence the part of the slides used for scoring. Moreover, difficulties can occur within the field of vision, such as previous biopsy-site related changes, biopsy-related hemorrhage, a substantial amount of extracellular mucin or the presence of infarcted and necrotic tissue. Lastly, resected tissue can contain ductal carcinoma in situ (DCIS) and other lesions. Recommendations regarding the assessment of fields containing the abovementioned difficulties are listed below.

### Blood vessels and nerves

Blood vessels and nerves are part of the TME and, therefore, attribute to the total stromal content. Still, in case large blood vessels are present in the microscopic field, it is preferred to choose another part of the tumor or, if no other appropriate area is available, to ignore it from scoring.

### Infiltration with inflammatory cells

Inflammatory cells within the microscopic field should be included in the stromal compartment for scoring, as these belong to the TME. However, this only applies to the infiltrate that is associated with the tumor. Lymphoid aggregates that are clearly separate from the tumor tissue should be neglected.

### Biopsy effects in resections

**Fig. 4:**
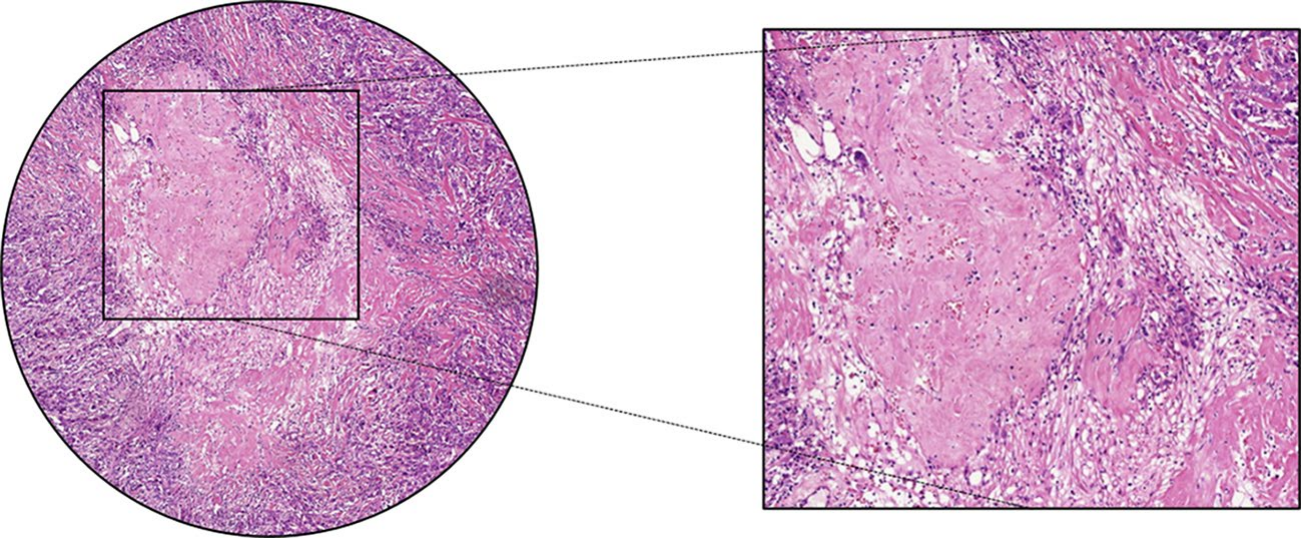
Circular annotation including a magnification of a primary breast tumor containing an area showing the characteristics of a biopsy effect (on the left side field view with × 10 objective). Note: the magnified image (right) was not used for TSR scoring, this was performed using the circular annotation (left)

Biopsy effects are often present in resection material, due to the relatively short interval between diagnosis and surgery (Fig. 4). The tissue reaction following a core needle biopsy procedure must not be mistaken for tumor-associated stroma, however, in daily practice this can be challenging. Typical features of a biopsy reaction are the presence of erythrocytes, a dense composition of the collagen in the specific area, presence of macrophages, hemosiderin, a track of a needle or the presence of a number of comparable areas, as often two or three samples are taken when performing a biopsy. In cases with a less recent biopsy, where tissue regeneration and scar formation are at a further stage, the differentiation between a biopsy effect and true tumor stroma may become problematic when no tumor cells are present. We advise to avoid areas suspected for biopsy trajectory in the scoring of the TSR.

### Mucus forming tumors

**Fig. 5:**
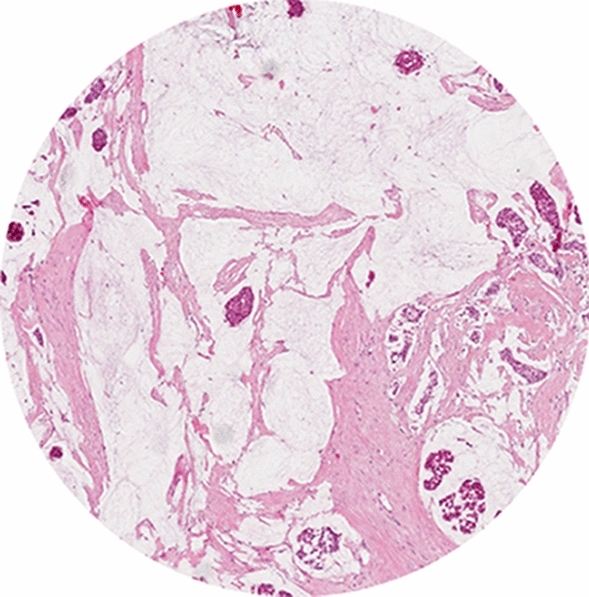
Digitized image of tumor tissue containing a possible difficulty for scoring: a stroma-high mucinous tumor (× 10 field view with digital microscope)

Some tumor types, such as mucinous carcinomas, contain a large amount of extracellular mucus. This is allowed to be present within the circular vision-site when assessing the TSR (Fig. 5). However, it is not part of the stromal compartment and should thus visually be excluded from the estimation of the stromal percentage.

### Diffusely infiltrating breast cancer

In rare instances, the diffusely infiltrative pattern mainly of invasive lobular carcinomas can occur as single tumor cells spreading between adipocytes. Even in diffusely infiltrating breast cancers, a part of the tumor possesses contiguous stroma. If this part is not present in a core biopsy, TSR scoring is not feasible. In such surgical specimens, one should look for the part of the tumor where connective tissue stroma is present and TSR scoring should be performed there.

### Multifocal breast cancer

There is very little experience regarding TSR scoring in multifocal breast cancers. In multifocal breast cancers, not only intratumoral, but also intertumoral heterogeneity may be present: such cases may be heterogeneous regarding immunophenotype and very rarely even regarding histological type. We suggest to select the tumor focus with the most abundant stroma for TSR scoring in surgical specimens. As a consequence of the intertumoral heterogeneity, in such cases significant differences may occur between the TSR scores of core biopsy and surgical specimen.

### Necrotic tissue

Stromal compartments of the tumor that include necrosis are ineligible for the assessment of the TSR. Moreover, areas that might indicate the formation of necrotic tissue should also be excluded from scoring and, similar to the mucinous parts, preferably be left out of the image field. If no other appropriate area is present on the tumor slide for scoring, the necrotic part should visually be excluded.

### DCIS and other in situ and benign lesions

DCIS consists of malignant cells that are not invading the surrounding stroma. Hence, these tumor cells should not be included in the TSR assessment and should be left out of the visual field. This is also applicable to other in situ and benign lesions. If these areas cannot be avoided, one should visually ignore them when assessing the TSR.

## Discussion

The TSR has proven its role as a prognostic parameter for patients with breast cancer, demonstrating that stroma-high tumors are associated with worse outcomes. The method of scoring the TSR is relatively easy, but demands a clear protocol to maintain the good interobserver variations that have been obtained in previous studies and to achieve that future research will use a uniform TSR scoring method, aiming toward implementation.

Several histological subtypes of invasive breast cancer are recognized, the most common being the NST. This histological type, on its own, is extremely heterogeneous. TSR investigations so far often mainly included the most common subtypes, e.g., NST breast carcinomas and invasive lobular carcinomas. Rare breast cancer types, like metaplastic carcinomas, have not been studied extensively for the TSR and, therefore, require additional research. Investigations specifically correlating the TSR to intrinsic subtypes have however been performed, especially for triple-negative breast cancer [6].

Assessment of histological markers is increasingly becoming a digital procedure. Digital slides have the advantages of preservation of stains’ quality for future research and facilitation of data exchange between different institutes for study or diagnostic purposes. Hence, digital image analysis (DIA) is becoming increasingly important for the assessment of tissue markers that can contribute to personalized medicine in terms of diagnosis and patient selection for treatment [37]. However, in some aspects, TSR scoring using digitized tumor material differs from the conventional microscopic method. As a result, a few different rules apply to the digital TSR scoring procedure.

The majority of the studies that have been performed to date include breast cancer resection material for the assessment of the TSR. However, to evaluate the effect of neoadjuvant treatment on the TSR or the predictive power of the TSR for a specific type of neoadjuvant treatment, it is necessary to make use of biopsy material acquired before the start of treatment. Biopsies have demonstrated to be of clinical importance in TSR scoring, not only to determine the prognostic value of the TSR in terms of survival (disease-free and overall) [38, 39] and relapse risk, but also to evaluate the predictive value of treatment outcomes with regard to histological parameters for pathological response [16, 40, 41].

There is an increased interest in evaluating the TSR in metastatic lymph nodes, in addition to the primary tumor. Previous studies have shown that the metastasizing process to the lymph nodes is a heterogeneous process and, therefore, the TSR scores of the primary tumor and the lymph nodes can be discordant [11]. However, estimation of the TSR on lymph node specimens in combination with the primary tumor can be of additional value in predicting breast cancer relapse. A similar outcome with regard to disease-free survival was seen for colon cancer [16]. It is, therefore, clinically valuable to assess the TSR on both the primary tumor and the lymph nodes, as it could strengthen the TSR and better patient selection for treatment.

A promising next step in the standardization of the TSR is the automated analysis of digital slides. In the method described in this paper, visual eyeballing was used to assess the TSR. However, in our experience, the tumors in which the stromal percentages of the fields of vision range around the cut-off of 50%, can lead to interobserver disagreements. Therefore, developing an automated scoring program to provide objective TSR scores is very promising. West et al*.* had already demonstrated the option of semi-automated point counting in breast cancer [42] and recently, the possibility of using computer-aided quantification and automatic deep learning has been evaluated in rectal cancer [43]. The latter showed that the TSR was still an independent prognostic parameter when analyzed automatically, similar to when it was scored by visual eyeballing in the same cohort of patients [44]. Moreover, the first step toward a deep learning model to quantify the TSR based on WSI of colorectal cancer tissue has also been taken [45]. Thus, further research into the automation of TSR analysis and deep learning models holds great opportunities [45, 46].

New projects are being introduced with the aim of implementing the TSR in current guidelines for improved risk stratification, which will most likely lead to better personalized treatment for patients with early-stage breast cancer. Part of this project will be the implementation of the TSR in the PREDICT model [47].

Before the TSR will be implemented in an online prediction tool such as the PREDICT model, it would be important to study the differences in TSR score when determined on biopsies and on resection material of primary tumors of the same patients and whether this is of influence on the prognosis. However, a preliminary experiment on a small cohort did not show a significant difference (J. Kulka, personal communication). For a larger future study in which the TSR score will be compared between biopsies and resection material, it will be important to note that neoadjuvant therapy can lead to changes in the composition of the stroma, resulting in the resection material of the tumor becoming unsuitable for TSR assessment. Therefore, the correlation between stroma status and prognosis should be evaluated in patients who did not receive neoadjuvant treatment.

In conclusion, visual evaluation of the TSR using simple H&E-stained sections has proven its prognostic value for breast cancer. Digital slides for either visual or automated analysis of the TSR evaluation offer future potential and further research in the field of automation is advised. Due to the relatively easy method of determining the TSR, implementation in routine pathological diagnostics would be the next step. This paper offers an extensive description of how the TSR is preferably scored for uniform data evaluation in future breast cancer studies. It addresses all major histological challenges within breast tumors that our group has encountered over the years and includes suggestions of how these can be overcome.

## Data Availability

Not applicable.

## Abbreviations

AI: Artificial intelligence
DCIS: Ductal carcinoma in situ
DIA: Digital image analysis
FFPE: Formalin-fixed paraffin-embedded
H&E: Hematoxylin and eosin
NST: No special type
TILs: Tumor-infiltrating lymphocytes
TME: Tumor microenvironment
TSR: Tumor-stroma ratio
WSI: Whole slide imaging
